# Accuracy of recording linear erosion using an unmanned aerial vehicle (UAV)

**DOI:** 10.1371/journal.pone.0329286

**Published:** 2025-09-08

**Authors:** Rebecca Hinsberger, Alpaslan Yörük

**Affiliations:** 1 Physical Geography and Environmental Research, Saarland University, Saarbrücken, Germany; 2 Hydraulic Engineering and Water Management, School of Architecture and Civil Engineering, University of Applied Sciences, Saarbrücken, Germany; Universidade Federal de Uberlandia, BRAZIL

## Abstract

Soil erosion is an ongoing environmental problem. To address this issue, calibrated erosion models are used to forecast areas vulnerable to erosion and to determine appropriate preventive measures. Model calibrations are based on erosion data recorded using different techniques such as photogrammetry from an unmanned aerial vehicle (UAV). In this study, the accuracy of the DJI P4 RTK UAV data was estimated for cropland boundary conditions. Ground heights of tilled and untilled arable land and standing water surfaces were determined using aerial surveys and compared to terrestrial surveys conducted on site. The results revealed that untilled soils can be accurately detected using a UAV, whereas the detection error rates of tilled soils were 2–3 folds higher. Additionally, the width and height of linear erosion tracks were measured and compared using aerial surveys and manual on-site measurements. The erosion width of the linear tracks was accurately recorded using a UAV whereas the erosion depth was underestimated by the digital elevation model (DEM) generated from UAV data.

## 1 Introduction

Erosion of agricultural land is an ongoing and highly discussed research topic. The collection of erosion data is important to calibrate and validate erosion models and to estimate the influence of erosion events on the environment [1,2]. To derive the spatial distribution and the volumetric quantity of erosion, erosion can be measured with direct contact techniques or with indirect non-contact techniques [3,4]. The direct method uses tools such as a metric ruler or rill meter. Especially in the early stages of erosion measuring, studies used mechanical profile meters to measure rill and gully cross-sections [5,6]. These tools are time-consuming due to intensive field work and expensive due to personnel costs [7]. According to Báčová et al. [3], the major disadvantage is the deformation of the soil surface during measurements and the limited resolution. The indirect method uses tools such as terrestrial laser scanners or photogrammetric methods that lead to fast data acquisition [3]. These methods require specific environmental and boundary conditions, such as weather and surface conditions. To evaluate the accuracy of these indirect methods, direct measurements were used for comparison in previous studies [7–9]. In addition, terrestrial laser data was compared to photogrammetric data [10–13] and the studies assume that photogrammetry is less costly but produces similar results in terms of accuracy.

Accuracy investigations of photogrammetry using UAVs were conducted in several studies. For the UAV ‘DJI P4 RTK’ used in this study, DJI [14] stated that the photogrammetry of the UAV can record surfaces with a vertical variance of ±1.5 cm (+1 ppm) and a horizontal variance of ±1 cm (+1 ppm). To achieve these values, optimal satellite configurations and optimal reception of real-time correction data are required [15]. For the accuracy of the UAV data, studies indicate variances of 2–3 cm for asphalt and grassland [15], observed vertical variances of 2 cm and horizontal variances of 1.2 cm on a solid surface [16], and suggested maximum vertical and horizontal variances of 4–5 cm for a facade [17]. To improve accuracy, some studies have focused on the usage of ground control points (GCP) [18–20]. These are linked in UAV images for additional georeferencing. Štroner et al. [20] indicate that horizontal measurements are adequate, but vertical variances are insufficient without GCP. Consequently, a combination of RTK (real-time kinematic) and GCPs provides the best results [20]. Martínez-Carricondo et al. [19] suggest one GCP at each edge of the investigation area to minimize altitude errors. For the evaluation of the errors, the root mean square error (RMSE) and the mean absolute error (MAE) are widely used statistical values. The RMSE is better for (normal) Gaussian errors and the MAE for Laplacian errors [21]. However, it is common to present both values and leave it up to the reader to choose which error value to use.

UAV-based photogrammetry presents a balance of resolution and efficiency and, consequently, a good compromise between field measurements and satellite-based remote sensing [22,23]. The photogrammetric method results in digital elevation models (DEM) and orthophotos with a high resolution of a few centimeters [22] and provides a good opportunity to measure soil surface changes [24].

Erosion structures have been investigated using different indirect methods, such as specifically designed kites [25], fixed-wing aircrafts [23,26], and UAVs [4,24]. These observation data were used to detect and monitor (gully) erosion [22,27,28]. [25] recorded different gully geomorphology and pointed out that the shadow of gullies negatively influences photogrammetric methods. Another study reported inaccuracies due to vegetation, water, and small-scale textures [29]. A study on the Chinese Loess Plateau [22] indicated the influence of different terrain characteristics on the accuracy of the UAV data.

This study focuses on evaluating the accuracy of the DEM derived from photogrammetric data collected by the DJI P4 RTK UAV for erosion surface conditions, specifically bare soil of croplands and linear erosion tracks. In order to apply the observed erosion quantity data in erosion model calibration or validation, it is crucial to estimate the errors present in the observational data. The surface structure is particularly important for this estimation [22], so the focus was on erosion surface conditions, such as bare soil with tilled and untilled surfaces. Both tilled and untilled surfaces are potentially present in the case of an erosion event. The use of UAV-based photogrammetry was considered suitable due to its capability to generate high-resolution DEMs necessary for studying soil microrelief. Aerial surveys were conducted on three bare soil fields with tilled and untilled soils as well as in an area with standing water and steep slopes. The latter test was used to assess how remaining water from a previous heavy precipitation event might affect the accuracy of erosion extent estimated by photogrammetry. The quality of these surveys was validated by comparing them to terrestrial measurements, as previously done in other studies [7–9]. The high geospatial accuracy of the selected UAV made it ideal for these comparisons.

Furthermore, the study aimed to examine the accuracy of linear erosion width and depth in UAV-based DEMs. Croplands spanning several hectares with erosion tracks were recorded using the UAV and the erosion tracks were measured using the direct method. By comparing the UAV-derived measurements of linear erosion tracks to those obtained with a metric ruler, the study assessed the accuracy of the UAV-generated DEM for erosion tracks, in particular. The objective of this part of the study was to investigate the accuracy and usability of the DJI P4 RTK for erosion investigations and identify potential uncertainties in erosion recording.

## 2 Materials and methods

**Fig 1:**
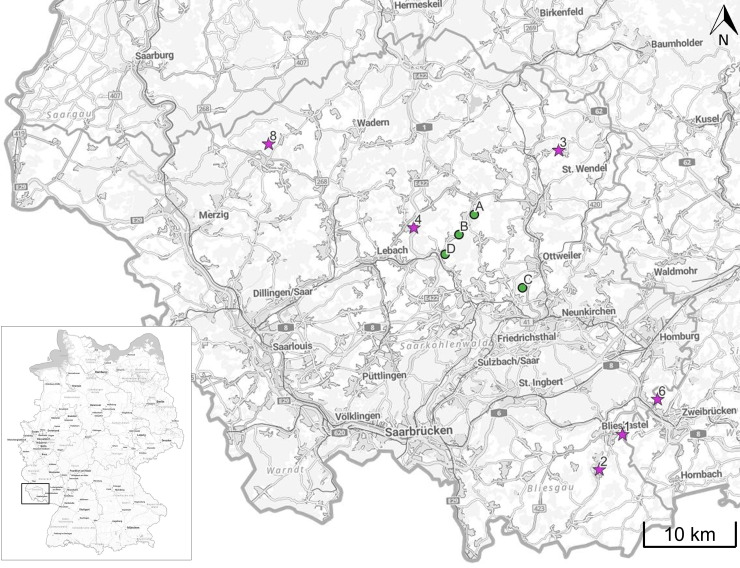
Overview of investigation areas. All areas are located in southwestern Germany, in the federal state of Saarland. Fields marked with green dots and labeled A–D are used for the investigations of different tillage and water surfaces (Section 2.2); Fields marked with purple stars and numbered 1–8 are used for linear erosion measurements (Section 2.3). Basemap: Reprinted from BKG under a CC BY license, with permission from BKG, original copyright 2025. © GeoBasis-DE/ BKG (2025) CC BY 4.0.

In this study, the accuracy of a UAV-generated DEM was investigated for tilled and untilled soil, as well as linear erosion tracks. Direct measurements were used as a benchmark. All investigated fields are located in southwestern Germany, in the federal state of Saarland. Two measurement series were conducted: i) To analyze the accuracy of the UAV-generated DEM due to tillage and water, four investigation areas were selected. These croplands were known by municipalities to experience erosion and are marked as Fields A–D in Fig 1; ii) To analyze linear erosion, six fields were selected. These fields were affected by real erosion events occurring over a period of two years and are marked as Fields 1–8 in Fig 1. Field 5 and 7 were excluded from this study because no manual measurements were conducted on these fields.

Prior to the surveys, permissions for the investigations were obtained from the tenant of each individual field.

### 2.1 General evaluation method

The UAV used in this study was a DJI Phantom 4 with real time kinematic (P4 RTK). The UAV is equipped with a built-in camera (1-inch CMOS sensor, effective pixel of 20 M, 24 mm wide-angle lens) and an RTK system for improved geospatial data [14]. The positioning precision was further enhanced by using a combination of the UAV and DJI D-RTK 2 mobile station. The output coordinate system for all aerial surveys was WGS84 with ellipsoidal heights. Since terrestrial surveys were conducted in the DHDN 3, Gauss-Kruger Zone 2 coordinate system (EPSG: 31466), the aerial surveys were transformed to this coordinate system and the German height system, DHHN2016.

All aerial surveys in this study were conducted in 2D mode, where images are recorded perpendicular to the ground. For the study on the water surface (Section 2.2), an additional survey in 3D mode was conducted. In 3D (multi-oriented) mode, the UAV initially records images in 2D mode and then takes additional images by rotating the camera gimbal by 30°. These additional images are recorded from four sides of the investigation area.

In the post-processing of the aerial surveys, the structure from motion (SfM) technique using Agisoft Metashape Professional software (Agisoft LLC, St. Petersburg, Russia) [30] was used. The photos taken by the UAV were aligned, and a dense cloud was built. From this dense cloud, a digital elevation model (DEM; referred to as UAV DEM) and an orthoimage were generated. The software settings were configured based on Agisoft’s specifications, matching those used in previous studies [20], and are presented in Table 1.

**Table 1:** Agisoft Metashape software settings used for the generation of the UAV DEM and orthoimages.

Setting	Value
Align Photos	
Accuracy	High
Key point limit	40,000
Tie point limit	8,000
Optimize Camera Alignment	Fit f, cx, cy, k1-4, p1-2
Build Dense Cloud	
Quality	High
Depth filtering	Aggressive
Build DEM	
Projection	Geographic
Source data	Depth maps
Quality	High
Interpolation	Enabled
Build Orthomosaic	
Surface	DEM
Blending mode	Mosaic (enable hole filling)

### 2.2 UAV accuracy due to tillage and water surfaces

**Fig 2:**
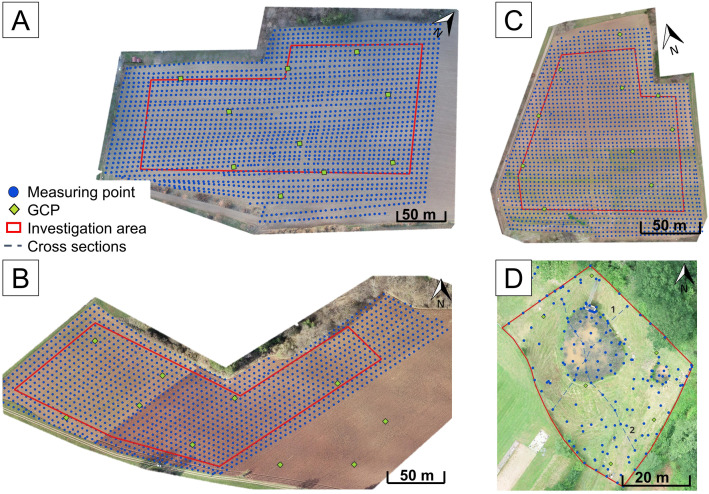
Investigation areas for the analysis of the influence of tillage and water surfaces. Investigated croplands (A, B, and C) and flood protection system (D) marked with the respective investigation area (red outline). Terrestrial measured points are marked as blue dots and the cross sections in Field D as blue dashed lines. Ground control points (GCP) used for the positioning of the UAV are marked as green diamonds. The orthophotos that serve as background map are the results of the aerial surveys.

To estimate the accuracy of elevation measurements using the UAV, aerial (indirect method) and terrestrial (direct method) surveys were carried out at different investigation fields. Elevation measurements were compared and analyzed to evaluate the UAV DEM accuracy. Three croplands with bare soil (Fields A–C in Fig 1 and Fig 2A–C) and one flood control reservoir with standing water (Field D in Fig 1 and Fig 2D) were selected for the study.

All fields were investigated in the spring. Field A was tilled (Fig 2A), Field B was half tilled/half untilled (Fig 2B), and Field C was completely untilled (Fig 2C). Vegetation was absent, except for a thin moss layer on parts of Field C. The fields ranged from 3 to 5 hectares in size, with steep slopes, in average between 12 and 18.5%.

To reduce errors caused by vegetation in neighboring areas, the perimeter of all investigated fields was buffered inside (outlined in red in Figs 2A–C).

An important quality factor for aerial surveys is reproducibility. Thus, the UAV investigations on cropland were performed twice consecutively in each field to examine reproducibility. Both flights were carried out in the 2D mode, with consistent settings and the usage of an RTK-fix throughout the entire flight. Further analyses, independent of reproducibility, were conducted using the data from the second aerial survey.

Additionally, 2–3 GCPs per hectare (marked with green diamonds in Fig 2) were recorded using the Global Navigation Satellite System (GNSS) receiver ‘Trimble R6’. This receiver achieved standard deviations of 1.4 cm in position in preliminary tests. According to literature (see Section 1), these GCPs can enhance UAV accuracy. To confirm this and analyze the impact, the post-processing of the aerial survey was conducted with GCPs to enhance accuracy in one analysis and without GCPs in another analysis.

Parallel to the aerial survey, all fields were surveyed terrestrially with the total station ‘Trimble S7’ approximately in a 5 m grid. In Fig 2, these terrestrial survey points are marked by blue dots in each investigation field and serve as a reference.

To verify the UAV DEM accuracy, the survey points from the total station were compared with corresponding points in the UAV DEM, with a total of 3470 points (1619 and 1851 points for untilled and tilled soil, respectively). Elevation data from the UAV DEM was extracted at the total station survey points using the point sampling tool in the geo-information system QGIS [31]. This method was also used in literature [7] and, therefore, used for all comparisons of point and DEM elevations.

The flood control reservoir area (Fig 2D) has an extension of 0.2 ha. The surface consists of grassland with varying slopes, as well as stagnant and turbid water areas. The grass was cut the day before the surveys to minimize vegetation interference. Some vegetation in the water areas was not removed, resulting in localized measurement errors in this area. Similar to the investigations on arable land, aerial and terrestrial surveys were conducted concurrently. UAV flights were conducted in both 2D and 3D modes. The data collected was used to compare the different flight modes. For this terrestrial survey, the total station ‘Trimble M3’ was used to measure points across the entire area at significant locations, around the water level, and at the ground of the water reservoir. Two cross-sections (indicated by blue dashed lines in Fig 2D) at the steep slope and water area of the reservoir were used to compare the terrestrial and aerial measurements in 2D and 3D modes.

Details of all study areas and flight metrics are provided in Table 2.

**Table 2:** Details of study areas and flight metrics for Fields A–D.

Field	A	B	C	D	
Mode	2D	2D	2D	2D	3D
DEM extent [ha]	10.35	12.58	8.5	3.57	5.57
Investigated area [ha]	1.92	3.09	3.05	0.2	
Investigated measuring points	893	1308	1269	182	
Flight date	19 March, 2021	02 March, 2021	22 March, 2021	25 June, 2021	
Flight height [m]	50	50	50	50	
Number of pictures	300	311	228	168	399
Overlapping [%]					
Horizontal	70	70	70	70	70
Vertical	80	80	80	80	80
Speed [m/s]	3.9	3.0	3.9	3.9	3.9
Obtained resolution UAV DEM [cm/px]	3.3	3.8	3.6	4.1	4.7

### 2.3 Analysis of linear erosion measurements

**Fig 3:**
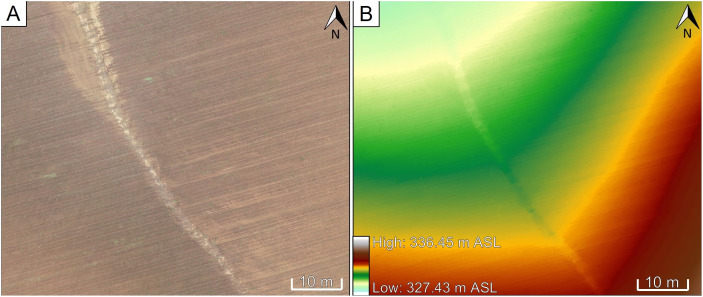
Recorded linear erosion track on Field 8. The erosion track is clearly visible on the orthoimage (A) and the DEM (B) generated using photogrammetric UAV data.

Measurements of eroded croplands are crucial for achieving the main objective of using erosion data from natural events for model calibration. In this study, six eroded croplands without vegetation were investigated over a period of two years. Aerial surveys were conducted using the DJI P4 RTK. The survey and photogrammetry processes in Agisoft Metashape Professional resulted in the generation of a DEM and orthoimage of the investigated area (see Section 2.1). Both grids have a resolution of a few centimeters that allows the clear visibility of linear erosion on both the orthoimage and the DEM (Fig 3).

Groove depth and width are essential measurements for accurately calculating erosion volume. To ensure quality control of these measurements, manual on-site measurements using a measuring stick were compared to the depth and width of grooves in the UAV DEM and orthoimage. The measuring points were located in six fields, with Field 1 being a potato field with bare soil and Fields 2, 3, 4, 6, and 8 being cornfields with bare soil or sparse vegetation. In total, 48 and 44 points for groove depth and width, respectively, were recorded. The precise location of the measuring points is essential due to the partly immense heterogeneity in different parts of the grooves. Thus, the positions of the on-site measurements were recorded using the GNSS receiver ‘Trimble R6’. As mentioned before, an accuracy of ≤1.4 cm of the position was possible. With the RTK system of the UAV, this point can be accurately represented in the UAV DEM with erosion. The groove width and depth were determined from cross sections in the DEM at the measuring points of the manual measurements.

## 3 Results and discussion

### 3.1 UAV-generated DEM accuracy due to tillage and water surfaces

The analysis of investigations on arable land with bare soil (Fields A–C) produced results regarding the i) reproducibility of UAV flights, ii) accuracy of the UAV DEM with and without GCPs, and iii) influence of tillage. Investigations of standing water in Field D produced results regarding iv) accuracy of recorded water surfaces and vegetation, and v) accuracy of photogrammetry in 2D and 3D modes.

#### Reproducibility.

For each of the investigation areas A–C, two consecutive flights were conducted and the photos from the UAV were used to create two DEMs without the use of GCPs. In Field A, the elevations in the DEM from the second flight are lower than those from the first flight. In Field C, the opposite is true, while in Field B, there are clusters where the elevations from either the first or second flight are higher. There is no discernible pattern, and tillage does not seem to have any influence. A comparison of all DEM grid cells (Field A: 21,044,504; Field B: 16,774,663; Field C: 20,115,771) shows a RMSE of 0.96 cm indicating a low scattering effect. Additional statistical values are available in Table 3, under the ‘reproducibility’ row. This error could potentially occur in all flights.

**Table 3:** Statistics of vertical error values for different accuracy analyses. Min = Minimum, Max = Maximum, Mean = Mean value, MAE = Mean Absolute Error, RMSE = Root Mean Square Error (all values in meter).

	Min	Max	Mean	MAE	RMSE
Reproducibility (Flight 1 – Flight 2, without GCP)	−0.7704	0.2346	0.0015	0.0119	0.0096
Accuracy UAV DEM					
Untilled – with GCP	−0.0664	0.0461	−0.0074	0.0145	0.0191
Untilled – without GCP	−1.1847	0.0325	−0.0161	0.0224	0.0499
Tilled – with GCP	−0.3367	0.0875	−0.0358	0.0390	0.0463
Tilled – without GCP	−0.2315	0.0540	−0.0645	0.0655	0.0752
Accuracy DEM1 (reg. authorities)					
Untilled	−0.4660	0.0940	−0.0785	0.0818	0.0980
Tilled	−0.2400	0.1080	−0.0587	0.0662	0.0817

#### Accuracy with and without GCPs.

To analyze the influence of GCPs on the accuracy of the DEM, post-processing of the second aerial survey was conducted with and without GCPs. For the DEM without GCPs, the same DEM used for the reproducibility analysis was used. A new DEM was processed for the DEM with GCPs. At the locations of the terrestrial survey points, both the UAV with and without GCPs were scanned for their elevations and compared to the directly measured terrestrial elevations. The results indicate that the DEM with GCPs aligns better with manual measurements compared to the DEM without GCPs. For untilled and tilled soil, the RMSE improved from 4.99 cm to 1.91 cm and from 7.52 cm to 4.63 cm, respectively. Additional statistical values are available in Table 3, under the ‘Accuracy UAV DEM’ rows. The negative mean value suggests that the elevations in the generated DEM tend to be higher than the directly measured elevations. Measurements with GCPs demonstrated the best performance in these investigations and were used for further analysis. This result is consistent with findings from previous studies [19,20]. In a study by Liu et al. [22], vertical RMSE values of 24.5 cm and 33.9 cm were observed for different locations using a microdrone md4−1000 and GCPs, which are higher than the values in this study. However, the mean values are 2 cm and 3 cm and show less deviation compared to the RMSE. The errors indicate that the mean value is similar and the values show a higher scatter in the study of Liu et al. [22]. The discrepancies may be attributed to the UAV used, study area, and flight height. In another study [19], the influence of the amount and distribution of GCPs was investigated using an octocopter. For a comparable stratified distribution with 2 GCPs/ha, they obtained a vertical RMSE of 4.3 cm. This value aligns with the results of this study. By using the DJI P4 RTK UAV that was also used in this study, Štroner et al. [20] observed mean deviations of −1.8 cm and standard deviations of 2.8 cm for vertical checkpoints in a rural area, further confirming the results of this study. The consideration of GCPs is very time-consuming [22], both for recording and marking the GCPs in the UAV photos. However, GCPs are important to consider when evaluating the accuracy of the UAV DEM.

The influence of tillage is analyzed in the following section.

#### Influence of tillage.

**Fig 4:**
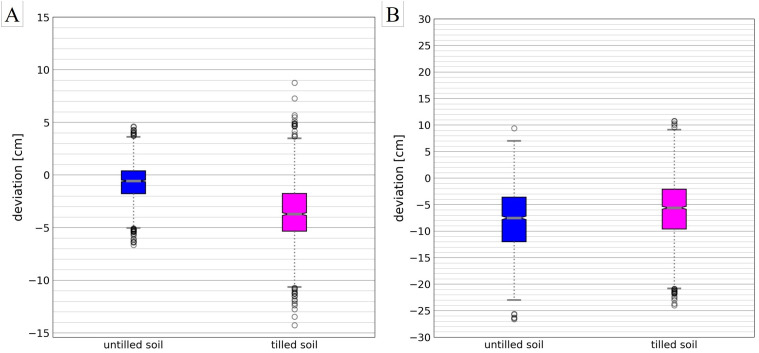
Calculated difference between terrestrial survey elevations and pursuant elevations from (A) UAV DEMs and (B) 1 m DEM provided by reg. authorities. There is a separate analysis for untilled (blue) and tilled (pink) soils. Negative values represent DEM elevations that are higher than terrestrial elevations.

Significant differences in accuracy were determined for measurements on tilled and untilled soils, indicating that the accuracy of UAV DEMs depends on tillage (Table 3, under the ‘Accuracy UAV DEM’ row). Fig 4A shows the differences between terrestrial survey elevations and UAV DEM elevations (with GCPs). The average absolute errors (MAE) were 1.45 cm and 3.90 cm, while the root mean square error (RMSE) values, indicating scattering, were 1.91 cm and 4.63 cm for untilled and tilled soils, respectively. For even and more solid surfaces, deviations suggested in the literature (see Introduction) were confirmed. The results are consistent with Khanal et al. [7], who used a DJI P4 (without RTK) and indicated varying accuracy for road and non-road surfaces, with a RMSE of 4.4 cm on even road surfaces and 19.7 cm on other areas. Uneven, soft soil surfaces led to lower quality results. These differences could be attributed to shades of field grooves, as observed in a previous study [25], and modifications in soft soil during manual total station measurements in the field. This modification can affect aerial surveys and lead to errors between aerial and terrestrial surveys. This main disadvantage of the direct method [10] could not be avoided. For accurately recording erosion on bare soil, better results can be expected when erosion tracks occur on untilled and even soil surfaces.

Furthermore, comparisons of survey points with pursuant points from a 1 m grid DEM provided by regional authorities were conducted (Table 3, under the ‘Accuracy DEM1, reg. authorities’ row, and Fig 4B). The 1 m DEM is often the only data available as a basis for modeling. This DEM was situated predominantly higher than the terrestrial survey points. In total, the differences between terrestrial survey points and the 1 m DEM are higher than the differences between terrestrial survey points and UAV DEM. One reason is the lower resolution of the 1 m DEM. This raster cannot display specific points or centimeter resolutions. Another important reason is the temporal gap between recording operations in 2016 for the 1 m DEM and March 2021 for Fields A–C. The tillage of arable land had less influence on accuracy differences from the 1 m DEM compared to the UAV DEM. Comparisons correspond better for tilled soil than untilled soil, but caps and fliers in the boxplots are similar (Fig 4B). Differences of up to 20 cm were observed, consistent with the accuracy statement of the regional authority [32].

#### Accuracy of water surfaces and vegetation.

**Fig 5:**
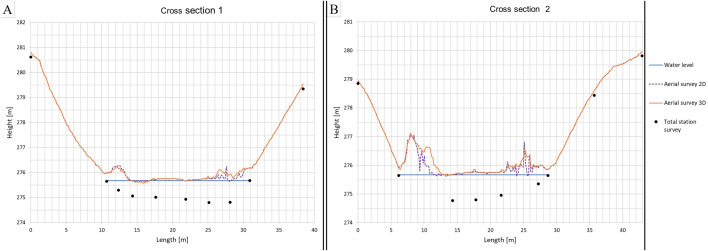
Comparison of measurements at cross sections in Field D. Measurements with the UAV in 2D mode are shown with a purple, dashed line, 3D mode with an orange line, and terrestrial survey points with black dots. Blue lines indicate the water level on the day of the surveys.

During the aerial survey of the flood control reservoir, the river water was stagnant, dark, and turbid. Comparisons of aerial and terrestrial survey points revealed that the UAV surveys of turbid water only captured the water surface. Vegetation on banks and foreland could cause measurement errors because only the surface of the vegetation was recorded. Fig 5 shows two examples of cross sections in the flood control reservoir. The position of the cross sections is shown in Fig 2D. Both cross sections show terrestrial survey points (black) and the water level height (blue line) on the day of the investigation. Aerial surveys in 2D (purple, dashed line) and 3D mode (orange line) are shown as well. In the middle of the water surface, aerial survey elevations align with the water surface. However, at the edges of the water surface, aerial survey elevations are influenced by vegetation. In the transition zone between the water and the bank, grass that could not be mowed resulted in measurement inaccuracies. These inaccuracies, caused by vegetation and water, are consistent with the results of previous studies [29].

In the context of erosion measurement, water surfaces, resulting from a precipitation event, can lead to errors. Depending on the water depth in erosion tracks, the erosion depth can be underestimated, because water surfaces are recorded during the aerial survey. As this circumstance can be a disadvantage of the method, it is recommended to conduct an aerial survey for the erosion record one day after the event to avoid any water residues. According to DWA [33], a maximum of 3 days should not be exceeded.

#### Accuracy 2D and 3D UAV mode.

For flat and lightly vegetated surfaces, the measurements of flights in 2D and 3D modes aligned well (Fig 5). There were no significant differences in slope changes observed in either mode. However, dense vegetation in the water area resulted in different elevations in 2D and 3D mode measurements. In the 3D mode, measurements often displayed enveloping lines for the tallest vegetation, while the 2D mode showed fluctuations in vegetation heights. This discrepancy can be attributed to the number of images and different camera angles in the 3D mode. Therefore, the 2D mode is only suitable for soil surfaces lacking vegetation. The comparability of results between 2D and 3D modes is an advantage, as aerial surveys in 2D mode are less time-consuming for recording and post-processing when using GCPs.

The investigations and analyses conducted showed that using the DJI P4 RTK represents a good option for investigating arable land with centimeter accuracy. However, potential errors arising from water and vegetation must be excluded. Erosion typically occurs on bare soils without any vegetation to protect them from rainfall. Therefore, the negative influence of vegetation on accuracy may often be neglected. For bare and untilled soils, as they may occur during heavy precipitation periods, the study reveals an average absolute deviation of 1.5 cm. For a 20 m long and 1 m wide gully, this equates to an erosion volume of ± 0.3 m³ of soil and a mass of 390 kg (assuming a density of 1,300 kg/m³).

### 3.2 Analysis of linear erosion measurements

**Fig 6:**
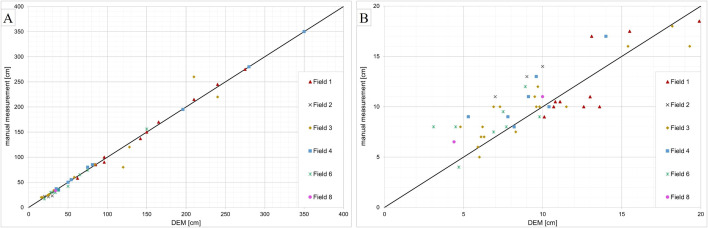
Comparison of DEM and manual measurements for (A) groove width and (B) groove depth.

For the quality assessment of erosion tracks, erosion fields were surveyed using the UAV one or two days after the heavy precipitation event. The groove width and depth were measured in the generated UAV DEM and manually with a measuring stick. Fig 6 shows a comparison of groove width (A) and depth (B) measurements obtained from the UAV DEM and the measuring stick (manual). The groove width is often easily recognizable in orthoimages and aligns well with manual measurements. Deviations in Field 3 occur because groove boundaries were washed out at the foot of the slope and partly covered with sediment. Thus, clear boundaries are difficult to define on the orthoimage. The manual rill width measurements range from 17 to 350 cm. In total, the indirect measurements correspond well for the rill width, with an RMSE of 10.8 cm for rills that are up to 350 cm wide. Additional statistical values are available in Table 4. The manual rill depth measurements range from 4 to 20 cm. A comparison of depth measurements shows that the UAV DEM underestimates groove depth with an RMSE of 2.13 cm for rills up to 20 cm (Table 4). However, Field 1 shows several measuring points that overestimated the groove depth. A possible reason for this discrepancy is the instability of the surface caused by furrows in the potato field. Measuring points on other fields also overestimated groove depth when the groove was washed out at the foot of the slope. In this case, the derivation depends on the individual. The narrower the groove, the greater the likelihood that the measuring points of the DEM would underestimate the groove depth. This could be caused by the shadowing of the groove [25]. Additionally, the reflection of gullies can be affected by their deep location and small terrain openness [22]. Aerial surveys have produced lower depth values, leading to an underestimation of the total groove depth and erosion volume, ultimately resulting in a minimum volume.

**Table 4:** Statistics for the difference between UAV and manual measurements of groove width and depth. Min = Minimum, Max = Maximum, Mean = Mean value, MAE = Mean Absolute Error, RMSE = Root Mean Square Error (all values in meter).

	Min	Max	Mean	MAE	RMSE
Rill width	−0.50	0.40	0.0037	0.0521	0.1083
Rill depth	−0.0490	0.0360	−0.0074	0.0170	0.0213

## 4 Conclusions

In this study, accuracy investigations were conducted using the DJI Phantom 4 RTK UAV. The results showed centimeter accuracy of the UAV-generated DEM for even surfaces and less accurate measurements for tilled soils. As erosion often occurs due to poor vegetation on arable land, vegetation does not adversely affect aerial measurements. Thus, aerial surveys of erosion using a UAV and photogrammetry are a useful method. For erosion measurements, the lack of shadowing and even surfaces without harrowed and plowed soil are advantageous. Comparisons of groove measurements showed that DEMs derived from recordings using a UAV reproduced groove width well. However, groove depth was underestimated compared to on-site direct measurements.

Collecting and recording natural erosion provides a foundation for the calibration of erosion models. With these models, appropriate simulations can be achieved to obtain information on areas at risk of erosion, which can be the basis for implementing countermeasures.

### Limitations

There are limitations in the application of UAVs for recording arable land that must be taken into account. UAV positions may have been adversely affected by wind gusts in some measurements. In addition, erosion often occurs during heavy precipitation. Recording erosion tracks is weather-dependent and can only be performed once the precipitation has stopped. It cannot be ruled out that changes in the fields were made between the erosion event and the measurements. The consideration of GCPs is very time-consuming [22]; both for recording and marking the GCPs in the UAV photos. Linear erosion can be affected by shadowing [25], deep location, and small terrain openness [22] (see Section 3.2). This study also demonstrated the impact of water and vegetation surfaces on aerial survey outcomes (see Section 3.1).

## References

[1] Malinowski R, Heckrath G, Rybicki M, Eltner A. Mapping rill soil erosion in agricultural fields with UAV‐borne remote sensing data. Earth Surf Processes Landf. 2022;48(3):596–612. doi: 10.1002/esp.5505

[2] Pineux N, Lisein J, Swerts G, Bielders CL, Lejeune P, Colinet G, et al. Can DEM time series produced by UAV be used to quantify diffuse erosion in an agricultural watershed?. Geomorphology. 2017;280:122–36. doi: 10.1016/j.geomorph.2016.12.003

[3] Báčová M, Krása J, Devátý J, Kavka P. A GIS method for volumetric assessments of erosion rills from digital surface models. European Journal of Remote Sensing. 2018;52(sup1):96–107. doi: 10.1080/22797254.2018.1543556

[4] Di Stefano C, Palmeri V, Pampalone V. An automatic approach for rill network extraction to measure rill erosion by terrestrial and low‐cost unmanned aerial vehicle photogrammetry. Hydrological Processes. 2019;33(13):1883–95. doi: 10.1002/hyp.13444

[5] Casalí J, López JJ, Giráldez JV. Ephemeral gully erosion in southern Navarra (Spain). CATENA. 1999;36(1–2):65–84. doi: 10.1016/s0341-8162(99)00013-2

[6] Casalí J, Loizu J, Campo MA, De Santisteban LM, Álvarez-Mozos J. Accuracy of methods for field assessment of rill and ephemeral gully erosion. CATENA. 2006;67(2):128–38. doi: 10.1016/j.catena.2006.03.005

[7] Khanal M, Hasan M, Sterbentz N, Johnson R, Weatherly J. Accuracy Comparison of Aerial Lidar, Mobile-Terrestrial Lidar, and UAV Photogrammetric Capture Data Elevations over Different Terrain Types. Infrastructures. 2020;5(8):65. doi: 10.3390/infrastructures5080065

[8] Pourali S.; Arrowsmith C.; Chrisman N.; Matkan A. Vertical accuracy assessment of LiDAR ground points using minimum distance approach. CEUR Workshop Proc. 2014, 1142, 86–96. In: Winter S and Rizos C, Editors. Research@Locate’14, Canberra, Australia, 07–09 April 2014, published at http://ceur-ws.org

[9] Soininen V, Hyyppä E, Muhojoki J, Luoma V, Kaartinen H, Lehtomäki M, et al. Accuracy comparison of terrestrial and airborne laser scanning and manual measurements for stem curve-based growth measurements of individual trees. Science of Remote Sensing. 2024;9:100125. doi: 10.1016/j.srs.2024.100125

[10] Castillo C, Pérez R, James MR, Quinton JN, Taguas EV, Gómez JA. Comparing the Accuracy of Several Field Methods for Measuring Gully Erosion. Soil Science Society of America Journal. 2012;76(4):1319–32. doi: 10.2136/sssaj2011.0390

[11] Glendell M, McShane G, Farrow L, James MR, Quinton J, Anderson K, et al. Testing the utility of structure‐from‐motion photogrammetry reconstructions using small unmanned aerial vehicles and ground photography to estimate the extent of upland soil erosion. Earth Surf Processes Landf. 2017;42(12):1860–71. doi: 10.1002/esp.4142

[12] Gómez-Gutiérrez Á, Schnabel S, Berenguer-Sempere F, Lavado-Contador F, Rubio-Delgado J. Using 3D photo-reconstruction methods to estimate gully headcut erosion. CATENA. 2014;120:91–101. doi: 10.1016/j.catena.2014.04.004

[13] Kaiser A, Neugirg F, Rock G, Müller C, Haas F, Ries J, et al. Small-Scale Surface Reconstruction and Volume Calculation of Soil Erosion in Complex Moroccan Gully Morphology Using Structure from Motion. Remote Sensing. 2014;6(8):7050–80. doi: 10.3390/rs6087050

[14] DJI. PHANTOM 4 RTK: User Manual, v2.4, 2021.07. 2021. https://www.dji.com/downloads/products/phantom-4-rtk

[15] Przybilla H-J, Bäumker M. Untersuchungen zur Qualität des Realtime Kinematic GNSS Systems der DJI Phantom 4 RTK Quality control of the realtime kinematic GNSS systems of DJI Phantom 4 RTK. Conference Paper, 40. Wissenschaftlich-Technische Jahrestagung der DGPF in Stuttgart – Publikationen der DGPF, 2020, Band 29, 2020. pp. 47–61.

[16] DroneDeploy; DJI; Trimble. Measurement Accuracy of the DJI Phantom 4 RTK & Photogrammetry. 2019. https://www.dronedeploy.com/resources/ebooks/measurement-accuracy-dji-phantom-4-rtk-drone-photogrammetry/

[17] Taddia Y, González-García L, Zambello E, Pellegrinelli A. Quality Assessment of Photogrammetric Models for Façade and Building Reconstruction Using DJI Phantom 4 RTK. Remote Sensing. 2020;12(19):3144. doi: 10.3390/rs12193144

[18] Forlani G, Dall’Asta E, Diotri F, Cella UM di, Roncella R, Santise M. Quality Assessment of DSMs Produced from UAV Flights Georeferenced with On-Board RTK Positioning. Remote Sensing. 2018;10(2):311. doi: 10.3390/rs10020311

[19] Martínez-Carricondo P, Agüera-Vega F, Carvajal-Ramírez F, Mesas-Carrascosa F-J, García-Ferrer A, Pérez-Porras F-J. Assessment of UAV-photogrammetric mapping accuracy based on variation of ground control points. International Journal of Applied Earth Observation and Geoinformation. 2018;72:1–10. doi: 10.1016/j.jag.2018.05.015

[20] Štroner M, Urban R, Reindl T, Seidl J, Brouček J. Evaluation of the Georeferencing Accuracy of a Photogrammetric Model Using a Quadrocopter with Onboard GNSS RTK. Sensors (Basel). 2020;20(8):2318. doi: 10.3390/s20082318 32325692 PMC7219658

[21] Hodson TO. Root-mean-square error (RMSE) or mean absolute error (MAE): when to use them or not. Geosci Model Dev. 2022;15(14):5481–7. doi: 10.5194/gmd-15-5481-2022

[22] Liu K, Ding H, Tang G, Na J, Huang X, Xue Z, et al. Detection of Catchment-Scale Gully-Affected Areas Using Unmanned Aerial Vehicle (UAV) on the Chinese Loess Plateau. IJGI. 2016;5(12):238. doi: 10.3390/ijgi5120238

[23] D’Oleire-Oltmanns S, Marzolff I, Peter KD, Ries JB. Unmanned Aerial Vehicle (UAV) for Monitoring Soil Erosion in Morocco. Remote Sensing. 2012;4(11):3390–416. doi: 10.3390/rs4113390

[24] Eltner A, Baumgart P, Maas H, Faust D. Multi‐temporal UAV data for automatic measurement of rill and interrill erosion on loess soil. Earth Surf Processes Landf. 2014;40(6):741–55. doi: 10.1002/esp.3673

[25] Giménez R, Marzolff I, Campo MA, Seeger M, Ries JB, Casalí J, et al. Accuracy of high‐resolution photogrammetric measurements of gullies with contrasting morphology. Earth Surf Processes Landf. 2009;34(14):1915–26. doi: 10.1002/esp.1868

[26] Peter KD, d’Oleire-Oltmanns S, Ries JB, Marzolff I, Ait Hssaine A. Soil erosion in gully catchments affected by land-levelling measures in the Souss Basin, Morocco, analysed by rainfall simulation and UAV remote sensing data. CATENA. 2014;113:24–40. doi: 10.1016/j.catena.2013.09.004

[27] Maugnard A, Cordonnier H, Degre A, Demarcin P, Pineux N, Bielders CL. Uncertainty assessment of ephemeral gully identification, characteristics and topographic threshold when using aerial photographs in agricultural settings. Earth Surf Processes Landf. 2014;39(10):1319–30. doi: 10.1002/esp.3526

[28] Zhang S, Li F, Li T, Yang J, Bu K, Chang L, et al. Remote sensing monitoring of gullies on a regional scale: A case study of Kebai region in Heilongjiang Province, China. Chin Geogr Sci. 2015;25(5):602–11. doi: 10.1007/s11769-015-0780-z

[29] Cook KL. An evaluation of the effectiveness of low-cost UAVs and structure from motion for geomorphic change detection. Geomorphology. 2017;278:195–208. doi: 10.1016/j.geomorph.2016.11.009

[30] Agisoft LLC. Software Agisoft Metashape Professional, Version 1.7.1, St. Petersburg, Russia (n.d.).

[31] QGIS. Software QGIS, Version 3.26.2. n.d.

[32] LVGL Landesamt für Vermessung, Geoinformation und Landentwicklung. Digitale Geländemodelle Digital elevation models. 2019. [cited 12.01.2022]. https://www.saarland.de/lvgl/DE/themen-aufgaben/themen/geotopographie/digitalegelaendemodelle/digitalegelaendemodelle.html

[33] DWA Deutsche Vereinigung für Wasserwirtschaft Abwasser und Abfall e. V. DWA-Regelwerk, Merkblatt DWA-M 921. Bodenerosion durch Wasser – Kartieranleitung zur Erfassung aktueller Erosionsformen Soil erosion by water – Mapping guideline to record current erosion forms., Entwurf. 2020. Druckhaus köthen GmbH & Co KG. Hennef. ISBN: 978-3-88721-853-9.

